# The relationship between cumulative family risk and athlete burnout among Chinese college athletes: the mediating role of negative affect

**DOI:** 10.3389/fpsyg.2023.1251663

**Published:** 2023-10-16

**Authors:** Rui Li, Xujuan Guo, Yuqian Liang, Yalun An, Maoyao Xu, Biao Zhang

**Affiliations:** ^1^School of Education, Beijing Sport University, Beijing, China; ^2^China West Normal University, Nanchong, China; ^3^School of Social Development and Public Policy, Beijing Normal University, Beijing, China; ^4^School of Psychology, Beijing Language and Culture University, Beijing, China

**Keywords:** athlete burnout, cumulative family risk, negative affect, Chinese college students, mediating

## Abstract

**Introduction:**

Burnout of college athletes affects the sports performance of college athletes, etc., and plays an important role in the career development of college athletes. China attaches great importance to the mental health development of college athletes, but the influence mechanism of college athlete burnout has not received attention. This study explored the relationship between cumulative family risk, negative affect, and athlete burnout in college athletes.

**Methods:**

Data on 824 college athletes from more than 40 universities in China were collected through convenient sampling, using questionnaires including Cumulative Family Risk Scale, Athlete Burnout Questionnaire, and Negative Affect Scale.

**Results:**

The results of the structural equation model show that cumulative family risk has a significant positive effect on college athlete burnout. Cumulative family risk has a significant positive effect on the negative affect of college athletes. Negative affect also plays a significant mediating role in the relationship between cumulative family risk and college athlete burnout.

**Discussion:**

These results suggest that cumulative family risk directly or indirectly affects collegiate athlete burnout.

## 1. Introduction

Burnout is a psychophysiological state caused by prolonged cognitive activity, which manifests itself in terms of subjective feelings, behavioral performance and physiological changes (Marcora et al., 2009; Van Cutsem et al., 2017). According to the Cognitive-Affective Model, burnout is caused by stress, emphasizing that burnout is a negative response to stress (Smith, 1986). From a special point of view, athlete’s burnout refers to a syndrome characterized by a decreased sense of achievement, emotional or physical exhaustion and negative evaluation of sport (Raedeke, 1997). In other words, it is a psychological function that occurs when athletes’ internal resources are continuously depleted without timely replenishment in response to internal and external stressors (Zhang et al., 2015). Burnout not only negatively affects an individual’s physical and mental health (Wright et al., 2008; Marcora et al., 2009; Van Cutsem et al., 2017), but also interferes with one’s cognitive activity processes and cognitive functioning (Van der Linden et al., 2003; Boksem et al., 2005; Kato et al., 2009; Wascher et al., 2014; Möckel et al., 2015). Specifically, fatigued individuals have difficulty maintaining attention on the task at hand and are susceptible to distractions from extraneous stimuli in the environment (Van der Linden and Eling, 2006), which may lead to a decrease in task performance levels (Lorist et al., 2005). Burnout shows itself as a reduced level of effort to the task at hand, or even exhaustion and an aversion to continuing the current activity (Wright and Stewart, 2012). Besides, it also leads to an increased risk of making mistakes (McCormick et al., 2012). Psychological burnout in college athletes could adversely affects their training variables such as training quality and competition results, as well as psychological variables such as motivation to achieve, and even affects their academic performance, social practice and other activities (Zhang and Zhang, 2010). Moreover, the greater the likelihood that college athletes experiencing cumulative family risk would develop adverse psychological and behavioral effects, the more likely they may be to develop psychological burnout (Evans et al., 2013). Negative affect may play an important role in this process, as they are closely related to individual mental problems and physical and mental health (Turner et al., 2020), and are also susceptible to environmental influences. Therefore, it is worthwhile to explore the burnout of college athletes and its formation mechanism with family cumulative risk, as well as the role of negative emotions in it.

### 1.1. College athletes’ cumulative family risk and athlete burnout

Family risk refers to the various factors that an individual encounters in the family microsystem that may increase their adverse development (Bronfenbrenner, 2005). Individuals who have experienced multiple risks are more likely to meet psychological blocks than those who experienced a single risk (Doan et al., 2012). Meanwhile, the different risk factors do not act in isolation, but rather exhibits greater “co-occurrence” and are more threatening to an individual’s mental health (Evans et al., 2013). The Cumulative Risk Model indicates that multiple risk factors in the household have a much greater impact on individuals than a single risk factor, i.e., the more risk factors accumulated in the household, the more individuals are likely to be affected (Evans et al., 2013; Luo et al., 2017). The factors of the cumulative risk index in the family included three main aspects: risk of family structure (divorced, reconstituted, single parent), risk of insufficient family resources (low social and economic class, low parental education, low family income) and risk of the family atmosphere (low family adaptability and high conflict; Buehler and Gerard, 2013).

Individuals who experience multiple risk factors early in life have more undesirable developmental outcomes than those who experience a single risk (Evans et al., 2007). Cumulative ecological risk positively predicts psychological burnout in college athletes (Gruhn et al., 2016). Cumulative risk factors in the family are found to prevent individuals from perceiving the purpose, value and meaning of their lives as they grow up, making them vulnerable to feeling confused and uninterested in life (Xu et al., 2020). In particular, family risk factors could have a negative impact on an individual’s mental health, positively predicting mental health problems such as anxiety, depression levels, and self-injurious behaviors (Xiong et al., 2020). Several studies have confirmed that cumulative family risk negatively predicted individuals’ positive emotions while positively predict individuals’ negative effects (Doan et al., 2012; Lucio et al., 2012). The more cumulative risk factors there are, the more pronounced the tendency for individuals to decline in academic ability and achievement (Evans et al., 2013). It has been affirmed that learning fatigue in higher vocational students is negatively influenced by various external environmental risk factors, showing a cumulative effect (Chen et al., 2022). The higher the number of cumulative risks, the more likely learning fatigue is to occur (Gao, 2017). In addition, the Cross-over Model also demonstrated (Frone et al., 1992) that work–family conflict could cause psychological distress in working and living, resulting in feelings of anxiety, frustration, fatigue and incompetence. Therefore, these persistent negative effects could cause burnout. Thus it could be seen, that there is a strong relationship between cumulative family risk and burnout, so we propose the hypothesis: cumulative family risk positively predicts burnout in college athletes (H1). Although a relationship between cumulative family risk and burnout in college athletes has been established, the underlying mechanisms explaining this relationship are unclear.

#### 1.1.1. The mediating role of negative affect

Negative affect means a comprehensive generalization of an individual’s subjective experience of unpleasant or miserable emotions. Individuals with negative affect are accompanied by stressful reactions, miserable expressions, unpleasant experiences or feelings, and exhibited anxiety, depression, and tension (Watson et al., 1988; Rubaltelli and Pittarello, 2018). Negative affect increases the risk of occurrence of some negative events (Wan et al., 2015; Lew et al., 2019; Wang et al., 2020), causing individuals to behave badly (Rawson et al., 1994).

The cumulative family risk is significantly and positively associated with negative effects among college students (Zeng et al., 2008). Studies have acknowledged that one-third of children in low to medium-income families had poor cognitive and social-emotion development (McCoy et al., 2016). At the same time, the family’s social and economic disadvantage are positively related to juvenile delinquency (Savolainen et al., 2018). In addition, parents in poor families generally have lower levels of education (Burlaka et al., 2014). The less parent–child communication, the lower parental involvement (Asfour et al., 2017). The mothers’ greater use of negative parenting styles (e.g., psychological control) and maternal anxiety parenting (Weitkamp and Seiffge-Krenke, 2019) make children in poverty more likely to experience negative affectual problems such as depression and anxiety (Burlaka et al., 2017). Cumulative family risk extensively influences adolescents’ internalizing problems (Buehler and Gerard, 2013) and have a positive predictive effect on individuals’ negative affect.

Many stress-related factors, such as stress perception, trait anxiety, and role conflict, are all valid predictor variables of sports burnout (Capel et al., 1987; Vealey et al., 1992; Kelley, 1994). That is, stress-induced negative affect would lead to burnout (Smith, 1986; Silva, 1990; Coakley, 1992). From a fundamental internal psychological perspective, the creation of individual negative emotions, which are the unmet basic needs, lead to burnout (Quested and Duda, 2011). Furthermore, positive emotions are significantly and negatively correlated with burnout. With more optimistic feelings, individuals feel less stress and therefore less burnout (Gustafsson and Skoog, 2012). Negative effects on athletes have been shown to positively predict burnout (Strümpfer, 2003; Steensma et al., 2007). Accordingly, this study hypothesizes that: negative effects played a mediating role in the prediction of cumulative family risk on burnout in college athletes (H2).

China regards building a sports-powerful nation as a core value criterion and strongly advocates the integration of sports and education, and college athletes play an important role in promoting the physical fitness and healthy growth of Chinese students (An et al., 2022). College athletes, as an important resource and reserve force for sports or school sports, has not received much attention. So it is necessary to strengthen and deepen the research on the mental mechanisms of college athletes. Previous research has explored that social support could restrain or prevent Athlete burnout through mental toughness and sports motivation base on Chinese samples (Shang and Yang, 2021). Relationship maintenance strategies negatively predicted burnout in Chinese adolescent athletes (Fan et al., 2023). Adaptive perfectionism negatively predicted burnout in Chinese adolescent athletes (Chen et al., 2008).

In conclusion, current research rarely explore the mental mechanisms of Chinese college athletes. To contribute more information, this study aims to verify the correlation between cumulative family risk, negative affect and athlete burnout among college athletes base on previous research and the reality of Chinese college athlete burnout. Also, we aim to explore the mediating role of negative effects between cumulative family risk and athlete burnout among college athletes. The findings of this study are expected to provide some basis for increasing support for college athletes in multi-aspect, maintaining physical and mental health, and reducing cumulative family risk and college athlete burnout.

## 2. Methods

### 2.1. Participants and procedure

In this study, more than 40 colleges and universities in eight provinces in China were selected by convenience sampling method, including Beijing and Sichuan provinces; and college athletes majoring in sports in each college and university were selected as the research subjects by whole population sampling method. Four researchers were responsible for liaising with the universities. One liaison officer was chosen in each university to be responsible for the distribution of questionnaire links. Eight hundred and twenty-four subjects (aged 17–30 years, M = 21.00 years, SD = 2.31 years) were randomly recruited by a convenience sampling method. Among them, 456 were male and 368 were female. The research covered university PE majors, both undergraduate and postgraduate, in the three major sports (basketball, football and volleyball), swimming, ice-snow sports and aerobics. Of these students, 17.0% were freshmen, 30.0% were sophomores, 23.7% were juniors, 12.0% were seniors and 17.4% were postgraduates.

### 2.2. Measures

#### 2.2.1. Cumulative Family Risk Scale

In this study, the Cumulative Family Risk Scale (CFRS) consists of four-dimensional subscales with a total of 47 entries. The family financial stress scale is translated and revised by Wang et al. (2010), with 4 items, such as “My family does not have enough money to buy the food I like” and “My family does not have enough money to buy clothes.” The study uses the family adaptability scale, a revised version of the adaptability scale translated by Fei et al. (1991), with 16 entries. Questions, for example, “Family members do their best to support each other during difficult times.” “My family members prefer to discuss problems with friends than with family members.” and the like are asked. The family conflict scale in this study is a revised version of the family conflict factor scale translated by Fei et al. (1991), with 9 items, such as “Family members often quarrel” and “Family members rarely get angry with each other publicly.” The parental bordering instrument is the revised version of the parent–child relationship questionnaire translated by Zhang et al. (2011), with 18 items, e.g., “You talk well with your parents,” “You feel comfortable and natural when you express your feelings to your parents,” etc. All dimensions are scored on a 5-point Likert scale, except for the family conflict dimension, which is scored on a 2-point Likert scale. The scoring and coding method in this study is based on previous research (Wu, 2020), where subscale scores are calculated first and then dichotomously code according to the division boundaries. The Cronbach’s α coefficient for the total scale is 0.915. The Cronbach’s α coefficient for the financial stress questionnaire is 0.911. The Cronbach’s α coefficient for the family adaptability is 0.886. The Cronbach’s α coefficient for the family conflict is 0.617. The Cronbach’s α coefficient for the parent–child relationship is 0.956. The scale is analyzed for validity and its KMO is 0.908, indicating that the data from the questionnaire is suitable for factor analysis. Bartlett’s test results in a value of *p* < 0.05, which concludes that the questionnaire was valid.

#### 2.2.2. Athlete Burnout Questionnaire

The Athlete Burnout Questionnaire (ABQ), developed by Raedeke and Smith (2001) and revised by Sun and Zhang (2013), is used in this study. The questionnaire is divided into three dimensions, namely emotional/physical exhaustion, reduced sense of accomplishment and sport devaluation, with 15 items. For example, “I have done a lot of good things this season,” and “I am so tired from training that I do not have the energy to do anything else.” The questionnaire is scored on a 5-point Likert scale. Although the three dimensions of the questionnaire are all part of burnout. They are relatively independent and have different weights. Therefore, the three dimensions could not be summed up directly. In that case, the weighting formula establishes by Zhang et al. (2010) (Z weighted total score of burnout = Z reduced sense of accomplishment*0.47 + Z emotional/physical exhaustion*0.21 + Z sport devaluation*0.32) is used to obtain the weighted total score of burnout. In this study, Cronbach’s α coefficient for the total questionnaire is 0.922. The Cronbach’s α coefficient for emotional and physical exhaustion is 0.873. The Cronbach’s α coefficient for the reduced sense of accomplishment is 0.627 and the Cronbach’s α coefficient for the sport devaluation dimension is 0.845. The questionnaire is analyzed for validity and the KMO was 0.925. This indicates that the data from the questionnaire is suitable for factor analysis and Bartlett’s test results in a value of *p* < 0.05, which concludes that the questionnaire is valid.

#### 2.2.3. Negative Affect Scale

This study uses the subscales of the negative affect dimension of the Positive and Negative Affect Scale (PANAS) developed by Watson et al. (1988) and revised by Qiu et al. (2008). The scale consists of 9 items, such as “ashamed” and “sad.” The scale is scored on a 5-point Likert scale. In this study, Cronbach’s alpha coefficient for the total scale is 0.967. The scale is analyzed for validity and the KMO is 0.934, showing its data is suited to do factor analysis. Bartlett’s test results in a value of *p* < 0.05, which concluded that the scale is valid.

### 2.3. Data analysis

SPSS 25.0 is used to analyze the collected data in three steps. The first is to analyze the reliability of the three scales. Next is to carry on the common method bias test and the normal distribution test for factors of the cumulative risk of the family, psychological burnout, and negative affect among college athletes. The last is to analyze the descriptive statistics and correlation analysis for each variable. On this basis, the mediation effect model is further tested by estimating the 95% confidence interval of the mediation effect by 5,000 times sample sampling using Model4 in SPSS macro program PROCESS4.0.

## 3. Results

### 3.1. Common method bias

For possible common method bias in the data collected, the study uses the self-report method. The Harman single-factor test is used to verify for common method bias on the basis of procedural controls (e.g., anonymous filling and answering, reverse scoring of some entries, etc.; Zhou and Long, 2004). The exploratory factor analysis results in the extraction of a total of 10 factors with a characteristic root greater than one, with a maximum factor variance of 25.40% (<40%). Therefore, there is no significant common method bias in the measurement (Ding et al., 2020).

### 3.2. Normal distribution test for the three main variables

The test of normal distribution for family cumulative risk, negative affect, and athlete burnout is conducted, and it is found that the variables were basically acceptable as a normal distribution. Among them, the Kurtosis of cumulative family risk is −0.41, Skewness is 0.47. The Kurtosis of negative affect is −0.18, Skewness is 0.43. Kurtosis of psychological fatigue burnout is 1.48, Skewness is 0.67 (Table 1).

**Table 1:** Normal distribution test for the three main variables (*N* = 824).

Variables	Q1	Q2	Q3	Kurtosis	Skewness
Cumulative family risk	0.00	2.81	8.00	−0.41	0.47
Negative affect	1.00	2.32	5.00	−0.18	0.43
Athlete burnout	1.00	2.76	5.00	1.48	0.67

### 3.3. Mean number, standard deviation and correlation analysis of variables

Descriptive statistics tests and correlation analyses are conducted on cumulative family risk, negative affect, and athlete burnout. It is found that there was a two-by-two significant positive correlation between the three variables as shown in Table 2, indicating that athlete burnout of college students increased with the cumulative family risk and negative affect, and at the same time, negative affect increased with the level of their cumulative family risk.

**Table 2:** Mean number, standard deviation and correlation analysis of variables (*N* = 824).

Variables	*M*	*SD*	1	2	3	4	5
1. Age	21.00	2.31	1				
2. Gender	1.45	0.50	−0.07	1			
3. Cumulative family risk	2.81	1.74	0.05	0.06	1		
4. Negative affect	2.32	0.93	0.01	−0.05	0.12***	1	
5. Athlete burnout	2.76	0.67	0.08*	−0.05	0.12***	0.35***	1

***p < 0.001, **p < 0.01, *p < 0.05 the same below.

### 3.4. The correlation between cumulative family risk and athlete burnout: the mediating role of negative affect

The results of the correlation analyses met the test for mediating effects. Hence, this study adopts Model 4 in the SPSS macro program PROCESS4.0 and the non-parametric percentile Bootstrap method of bias correction (5,000 sample size, 95% confidence interval). This study has cumulative family risk as the independent variable, negative affect as the mediating variable, athlete burnout as the dependent variable and gender as the control variable. The regression results are shown in Table 3. As shown, the cumulative family risk significantly predicts athlete burnout (*B* = 0.09, *t* = 2.73, *p* < 0.01). With the addition of negative affect, the cumulative family risk still significantly predicts athlete burnout (*B* = 0.06, *t* = 1.71, *p* < 0.05). In addition, cumulative family risk could significantly predict negative affect (*B* = 0.12, *t* = 3.60, *p* < 0.01) while negative affect could significantly predict athlete burnout (*B* = 0.33, *t* = 9.05, *p* < 0.001).

**Table 3:** Test for the mediating role of negative affect (*N* = 824).

Predictor variables	Equation 1: athlete burnout			Equation 2: negative affect			Equation 3: athlete burnout		
	*B*	*SE*	*t*	*B*	*SE*	*t*	*B*	*SE*	*t*
Age	0.06	0.02	1.77	0.00	0.01	0.11	0.06	0.01	1.82
Gender	−0.06	0.07	−1.77	−0.05	0.06	−1.51	−0.05	0.07	−1.37
Cumulative family risk	0.09	0.03	2.73**	0.12	0.03	3.60**	0.06	0.03	1.71*
Negative affect							0.33	0.04	9.05***
*R^2^*	0.13			0.13			0.33		
*F*	4.69**			4.91**			24.33***		

**Figure 1:**
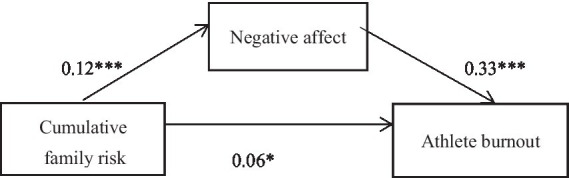
The mediating effect model. ***p < 0.001, **p < 0.01, *p < 0.05.

The results of the mediation analysis are shown in Table 4 95% confidence intervals for all three pathways for the total, direct and indirect effects all do not contain 0, indicating that the model for the mediating effect held. As followed: (1) cumulative family risk predicts athlete burnout directly (64.85% of the total effect); (2) cumulative family risk predicts athlete burnout through the mediation of negative affect (35.15% of the total effect; Figure 1).

**Table 4:** Breakdown table of the total, direct and mediating effect (*N* = 824).

	Effect	Boot standard error	Boot CI lower limit	Boot CI upper limit	Relative effect size
Total effect	0.68	0.20	0.29	1.08	1.00
Direct effect	0.44	0.19	0.07	0.82	0.65
The mediating effect of Negative affect	0.24	0.07	0.11	0.38	0.35

Boot SE, Boot LL CI and Boot UL CI, respectively, refer to the standard errors, lower and upper 95% confidence intervals of the indirect effects estimated by the bias-corrected percentile Bootstrap method.

## 4. Discussion

This study explores the correlation between cumulative family risk and athlete’s burnout, and the mediating role of negative affect on the relationship between cumulative family risk and athlete burnout in college athletes. The results showed that cumulative family risk is positively associated with negative affect and athlete burnout. Therefore, the hypothesis that cumulative family risk, negative affect and athlete burnout are related among college athletes is accepted. In addition, previous studies pay less attention to the psychological mechanisms of Chinese college athletes and the factors influencing athlete burnout. The findings would be helpful to explore the relationship between cumulative family risk, negative affect and athlete burnout in college athletes. These results provide potential intervention strategies to enhance burnout in Chinese college athletes.

### 4.1. Current situation

The results of the present study showed that Chinese college athletes burnout is at a high level (41.47 ± 10.12), which is consistent with previous research on Chinese professional athletes (Wang et al., 2023). Besides, college students who are not majoring in sports (16.96 ± 4.51) have lower scores in negative affect compared to the college athletes in this study (Tian and Zhang, 2009). Whereas increased levels of negative affect may lead to higher levels of burnout (Zhang and Hu, 2012). This also implies that college students majoring in sports are more likely to have high levels of burnout compared to college students not majoring in sports. Therefore, college athletes may face higher levels of burnout due to higher levels of negative affect. Higher levels of burnout in college athletes may be related to psychological factors such as emotional abuse (Humphreys et al., 2020), mental toughness (Ding et al., 2017), and emotional regulation flexibility (Cheng et al., 2014).

### 4.2. The relationship between cumulative family risk and athlete burnout

This study found that cumulative family risk is a significant positive predictor of burnout in college athletes, which is consistent with previous research (Zhan et al., 2023). Hypothesis 1 is verified. It has been suggested that prolonged or repeated exposure to risk leads to increased ‘wear and tear’ on the stress system, which increases the risk of burnout, and that individuals are exposed to an unpredictable and stressful risk environment that promotes the development of burnout (Ansell et al., 2012). Based on schema theory, the cumulative effect of various risk factors in the environment may cause individuals to experience adversity and frustration in the fulfillment of their core emotional needs, resulting in the emergence of a negative self-schema (Rupke et al., 2006), which may lead to burnout.

Individuals who are exposed to multiple risk factors often do not receive emotional support from family, school or peers, and lacked the resources to help themselves cope in the face of stress. Over time, individuals may deplete their resources, leading to burnout (Liu et al., 2023). Some studies have shown that in high-risk environments, higher vocational students receive less support are prone to impaired academic competence, have lower levels of academic achievement (Li et al., 2019), and in turn, burnout. Cumulative risk factors may negatively affect an individual’s internal self-schema, causing him or her to gradually develop lower self-esteem and self-efficacy, thus gradually weakening his or her motivation and efficacy in coping with stress, and eventually experiencing burnout (Li et al., 2019). Moreover, college students experiencing higher cumulative ecological risk are subjected to stressful life events and loss of resources, and neuroticism acts as a negative filter in daily life, amplifying adverse events and exacerbating stress and loss of resources (Semmer, 2006). College students in stressful environments may not have sufficient social support from parents or peers for high levels of achievement and are therefore vulnerable to experiencing burnout (Liu et al., 2023). Other possible explanations are that the more stressful the family conflict. The more likely people are to make negative and inappropriate evaluations of the event and themselves, the less mature coping styles, and more immature coping styles such as blaming, avoidance and denial, leading to burnout (Yuan et al., 2021; Zhan et al., 2023).

### 4.3. The mediating effect of negative affect

The mediation test finds that negative affect plays a mediating role between cumulative family risk and athlete burnout in college athletes, so hypothesis 2 is verified. The accumulated family risk could have a negative impact on an individual’s emotional development (Choi et al., 2019). Ego depletion theory identifies that human psychological resources are limited (Baumeister et al., 1998). When faces with multiple family risks, individuals mobilize internal resources to cope with the risks. If internal resources are not replenished in a timely manner, depletion and blockage of the retrieval process would result in impaired psychological health. There has been research finding that psychological capital moderated cumulative family risk and negative effects among university students. As the level of psychological capital increases, the negative prediction of cumulative family risk on negative affect among university students decreases, suggesting that psychological capital could buffer the negative affect of cumulative family risk on negative affect (Xiong et al., 2020). Other possible reasons for this are that cumulative family risk left children’s normal material and emotional needs unmet, which tends to keep them in a constant state of stress and anxiety and leads to emotional problems such as anxiety and stress (Evans and Kim, 2013).

It is found that athletes’ positive emotions are significantly negatively associated with all dimensions of burnout, with athletes in the low positive emotion group scoring higher on all dimensions of burnout than those in the medium positive emotion and high positive emotion groups (Gustafsson et al., 2010). Positive emotions predicted athlete enthusiasm to participate in sports, and athletes with more negative affect showed more emotional stamina exhaustion (Adie et al., 2008). A possible explanation for this is that a qualitative study of 10 top athletes who have competed internationally also find that negative affect is an important influence on mental toughness (Jones et al., 2007). Mental toughness enables athletes to cope better than their competitors with the stresses of competition, training and life and to persevere in the face of adversity. When there are fewer negative effects, athletes are less affected by symptoms of exercise fatigue (Jones et al., 2002; Madigan and Nicholls, 2017).

### 4.4. Research implication

The findings of this study have important implications for improving the burnout of college athletes. The negative impact of cumulative family risk on athletes is of concern, and studies have shown that increasing personal psychological capital (Zhang et al., 2010) and strengthening social support (Raikes and Thompson, 2005) could effectively reduce cumulative family risk. Consequently, schools and families should keep their eyes on the family risk situation of college athletes and do their best to assess and intervene. The role of negative affect in mediating the relationship between cumulative family risk and burnout in college athletes also illustrates that we could begin to reduce burnout by improving negative affect in college athletes. Studies have found that reducing stress levels (Folkman, 2008; Deng et al., 2012), implementing effective measures to ensure sleep quality (Zou et al., 2020) and reducing procrastination (Sirois and Pychyl, 2013) are effective ways to reduce negative affect in student-athletes. As a consequence, developing good personal habits and improving the ability of college athletes to cope with negative affect could help them to cope with negative affect caused by family risks and then reduce burnout.

## 5. Limitations and further direction

This study have achieved the expected results, but there are still some limitations in the current study. Firstly, the questionnaires use in this study all rely on self-report questionnaires, which may be potentially biased. Further research could consider using multiple methods or collecting multiple sources to reduce these biases. Secondly, the study provides useful information, but the cross-sectional design makes it difficult to clarify causal or tentative correlations between variables. Although we have tried to increase the sample size as much as possible, these limitations cannot be completely avoided. Thirdly, the sample size of this study is relatively small, and subsequent studies should increase the sample capacity to make the findings more representative. Lastly, future studies should consider the effect of sports performance when examining the mediating role of negative affect between cumulative family risk and athlete burnout to make the findings richer.

Therefore, future longitudinal studies focusing on this topic are needed.

## 6. Practical implications

The findings of this study have important implications for college athletes experiencing burnout. In the beginning, cumulative family risk is an important predictor of athlete burnout, and increasing psychological capital (Zhang et al., 2010) and strengthening social support (Raikes and Thompson, 2005) could effectively mitigate cumulative family risk. Next, based on the mediating role of negative affect in college athletes’ cumulative family risk and athlete burnout, we find that reducing college athlete stress levels (Folkman, 2008; Deng et al., 2012), implementing effective measures to safeguard sleep quality (Zou et al., 2020), and reducing procrastination (Sirois and Pychyl, 2013) are resultful ways to reduce college athletes’ negative affect. Finally, developing college athletes’ effective responses to chronic stress (Folkman and Lazarus, 1985) and increasing social support (DeFreese and Smith, 2013), in turn, prevent and treat college athlete burnout. In conclusion, controlling or reducing cumulative family risk and dissipating negative affect requires increased social, family, and school support, and giving college athletes ample time to rest and space to develop.

## 7. Conclusion

In summary, this study further expands the psychological mechanisms of Chinese college athletes and clarifies the current status of college athlete burnout. Besides, the correlations between cumulative family risk, negative affect and athlete burnout have been explored, as well as the mediating role of negative affect on the relationship between cumulative family risk and athlete burnout in college athletes. The results show that the cumulative family risk of college athletes is positively associated with negative affect and athlete burnout. Thereupon, the hypothesis that cumulative family risk, negative affect and athlete fatigue are related is correct. In order to motivate college athletes to rationalize athlete burnout, attention should be paid to alleviating college athlete burnout by implementing relevant interventions, and providing professional education and training and social support to college students.

## Data availability statement

The original contributions presented in the study are included in the article/supplementary material, further inquiries can be directed to the corresponding author.

## Author contributions

All authors listed have made a substantial, direct, and intellectual contribution to the work and approved it for publication.
